# Bacterial agents and antibiotic resistance in febrile neutropaenia in Africa: A systematic review and meta-analysis

**DOI:** 10.4102/ajlm.v14i1.2816

**Published:** 2025-08-26

**Authors:** Temitope O. Obadare, Adeyemi T. Adeyemo, Oluwaseun A. Ibrahim, Naheemot O. Sule, Mayowa M. Adeyemo, Olusegun I. Alatise

**Affiliations:** 1 Department of Medical Microbiology and Parasitology, Obafemi Awolowo University Teaching Hospitals Complex, Ile-Ife, Nigeria; 2 Department of Medical Microbiology and Parasitology, Faculty of Basic Clinical Sciences, Obafemi Awolowo University, Ile-Ife, Nigeria; 3 Department of Epidemiology, Faculty of Biostatistics and Occupational Statistics, McGill University, Montreal, Quebec, Canada; 4 Center for Clinical Epidemiology, Lady Davis Institute, Jewish General Hospital, Montreal, Quebec, Canada; 5 Department of Hematology, Pinderfields General Hospital, Mid Yorkshire NHS Trusts, Wakefield, United Kingdom; 6 Department of Surgery, Faculty of Clinical Sciences, Obafemi Awolowo University, Ile-Ife, Nigeria; 7 Department of General Surgery, Obafemi Awolowo University, Teaching Hospitals Complex, Ile-Ife, Nigeria

**Keywords:** febrile-neutropaenia, bloodstream-infection, bacterial agents, multi-drug resistance, Africa

## Abstract

**Background:**

Febrile neutropaenia (FN) is an oncology emergency, but there is a paucity of data on it in Africa.

**Aim:**

This study aimed to review and aggregate data on FN in the context of antibiotic resistance.

**Methods:**

Published original articles between 1991 and 2024 were systematically searched in Google Scholar, PubMed, and African Journals Online databases (grey literature excluded). ‘Febrile neutropenia’ was combined by Boolean terms ‘OR’ and ‘AND’ with individual countries for the searched terms. Data aggregation on bacteria isolates and antibiotics was done using Microsoft Excel.

**Results:**

Of 16 637 articles retrieved, 15 (from nine countries) with 1216 non-duplicate isolates were included in the analyses after exclusion of irrelevant and duplicate articles. There were 57.0% (698/1225) Gram-positive and 43.3% (527/1225) Gram-negative bacteria. Aggregated resistance to antibiotics for Gram-positive bacteria was 71.8% (163/227), for ampicillin, 74.3% (226/304), for cefoxitin, 64.1% (25/39), and 54.0% (47/87) for oxacillin, while that of Gram-negative bacteria was 35.5% (184/519) for ciprofloxacin, 60.6% (168/277) for ceftriaxone, 65.9% (89/135) for cefuroxime, and 38.2% (153/401) for imipenem. *Staphylococcus aureus* had 68.8% (22/32) resistance to oxacillin/methicillin and 10% (1/10) resistance to vancomycin. *Klebsiella* spp. was 50% (9/18) resistant to quinolones, 75.9% (22/29) resistant to third-generation cephalosporins, and 25.0% (4/16) resistant to carbapenems, while *Acinetobacter* spp. was 85.7% (6/7) resistant to gentamycin.

**Conclusion:**

This review highlighted the paucity of data and the emergence of multidrug resistance in FN in Africa. There is a need for antibiotic-resistance surveillance and antibiotic stewardship to optimise therapy in FN in Africa.

**What this study adds:**

To the best of our knowledge, this is the first systematic review of FN in Africa in the context of available laboratory resources across the African regions.

## Introduction

Cancer is one of the leading causes of morbidity and mortality globally and the fifth leading cause of mortality in Africa.^1^ According to Rajesh Sharma and colleagues using the GLOBOCAN 2020 estimate, about 1.1 million new cancer cases occurred in 2020, with over 700 000 mortalities in Africa.^2^ This figure might increase by 102% to 2.12 million annually by 2040, with an accompanying doubled mortality rate if nothing is done to reverse the trend.^3^ The low- and middle-income countries (LMICs) bear the brunt of cancer death, with overall 70% of mortality occurring in these regions because of delayed diagnosis, poor health infrastructure for cancer support, scarcity of oncology specialists, exorbitant cost, or lack of access to anti-cancer chemotherapy.^4^ In 2011, the Brazzaville Declaration on Non-communicable Diseases Prevention and Control in the World Health Organization African Region was adopted, where African countries acknowledged non-communicable diseases, including cancer, as public health issues of concern, but national cancer control plans have been poorly funded and coordinated.^5^

Neutropaenia is an absolute neutrophil count of less than 1.0 × 10^9^ cells/L in individuals of central European descent, or less than 1.2 × 10^9^ cells/L in African people.^6^ This is a common phenomenon in patients with cancer because of complications of cytotoxic chemotherapy, bone marrow metastatic replacement, and disruption of haematopoiesis by malignant cells. Febrile neutropaenia (FN) is defined as a single oral temperature greater than or equal to 101 °F (38.3 °C), or a temperature greater than or equal to 100.4 °F (38 °C) for at least an hour, with an absolute neutrophil count of less than 1500 cells/L.^7^ Febrile neutropaenia is an oncological emergency associated with sepsis, high treatment cost, treatment delay, and dose reduction, with potential compromise of cure in the patient.^8^ The severity and incidence of FN are dependent on various factors such as the content of the therapeutic chemotherapy, the cycle of therapy, age of the patient, type of malignancy, and other comorbidities of the patients.^9^ The FN annual incidence in the United Kingdom is 9.4 per 1000 oncology admissions.^10^ In the United States, the incidence of FN ranges from 7.83 cases per 1000 (solid tumours) to 43.3 cases per 1000 (haematological malignancies).^11^ The incidence of mortality is high in patients with FN, ranging from 50% to 75%.^12^

Early appropriate prophylaxis and empiric antibiotic use have significantly contributed to reduced morbidity and mortality in patients with FN.^8^ However, the emergence of multidrug-resistant (MDR) bacteria from misuse and overuse of antibiotics has reduced the effectiveness of broad-spectrum antibiotic use in patients with FN.^13^ It is documented that many antibiotics used in cancer patients are inappropriate and do not follow evidence-based standard guidelines.^14^ Moreover, the increased frequency and duration of contact with healthcare settings, breached anatomical barriers, and invasive procedures predispose patients with FN to healthcare-associated infections, which are mostly MDR pathogens, lower the survival rate in patients with FN.^13^

The World Health Organization predicts that the incidence of malignancy would increase by 48% by 2030 from the previous survey in 2018 in LMICs. This estimate could be less than the actual value, as there is an underestimation of cancer in Africa because of diagnostic capacity and cancer reporting.^15^ This would invariably increase the number of patients with FN in Africa. Unlike the developed countries, there is a dearth of data on the epidemiology of bacterial pathogens implicated in FN and their antibiotic susceptibility in Africa, which is responsible for the lag in the development of treatment protocols in many healthcare facilities in many African countries. In addition, the role of MDR, as well as its implication in FN, has not been well investigated, despite the high burden of infections by MDR pathogens in Africa, with challenges of poor infection prevention and control practices, indiscriminate antibiotic use, as well as inadequate diagnostic infrastructure. This study aimed to review the studies on FN conducted in Africa, and aggregate the data to document the patients’ clinical parameters, implicating bloodstream bacteria pathogens and their pattern of antibiotic resistance.

## Methods

This systematic review was performed according to the guidelines provided by the Cochrane Handbook for Systematic Reviews of Interventions and reported by the Preferred Reporting Items for Systematic reviews and Meta-Analyses guidelines.^16^

### Data sources and search strategy

Google Scholar, PubMed, and African Journals Online Library databases were systematically searched using relevant keywords (and indexed words) combined by Boolean terms ‘OR’ and ‘AND’ to form a final search strategy. The search term for Google Scholar [‘Febrile neutropenia OR neutropenia sepsis AND antibiotic OR antibacterial OR antimicrobial AND sensitivity OR resistance OR susceptibility AND the name of the individual African countries’], PubMed [‘Febrile neutropenia OR neutropenia sepsis AND the name of the individual African countries’], African Journals Online Library [‘Febrile neutropenia OR neutropenia sepsis AND the name of the individual African countries’] were tailored to each database, with year of publication restrictions from January 1991 to December 2024 for optimised performance at pulling out original articles published in Africa. Moreover, the references of the identified studies were hand-searched for other relevant studies not pulled by the searched terms from the databases.

### Selection criteria

Studies included in the synthesis of this systematic review were original observational studies published in peer-reviewed journals conducted in Africa on FN (or infection in oncology patients with FN components), which included a minimum of blood culture results with or without susceptibility pattern, with availability of the full texts. Review articles, letters, editorials, case reports, case series, conference summaries/abstracts, clinical trials and articles not published in the English language were excluded from the synthesis of this review. These selection criteria were predetermined by all the authors before the search. The study’s validity was ensured by strict compliance with the determined selection criteria.

### Study identification and article selection

**FIGURE 1:**
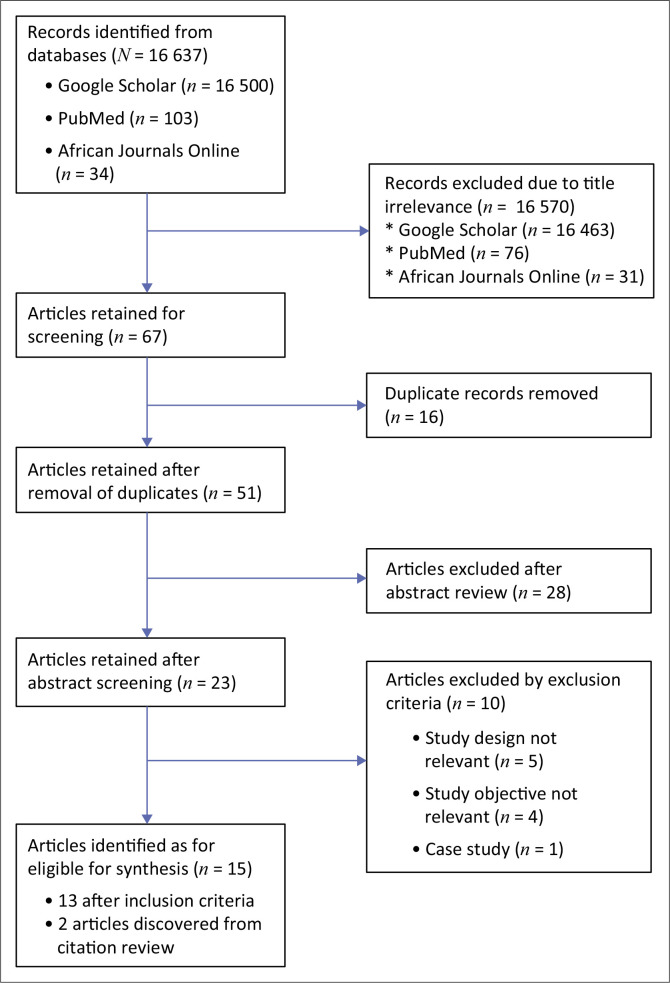
Preferred Reporting Items for Systematic reviews and Meta-Analyses flowchart describing literature search and identification of articles on febrile neutropenia in Africa, January 1991 – December 2024 for synthesis.

A total of 16 637 articles were pulled out by the search engines from the searched databases. Upon screening by titles, 16 570 were found to be irrelevant based on the predetermined parameters for this study. The remaining 67 articles were screened using Mendeley Citation Manager software (Elsevier, Amsterdam, Netherlands), and 16 were excluded for being duplicates. Twenty-eight articles were further excluded after screening of the abstracts and subsequent sorting based on inclusion criteria by two independent reviewers (T.O.O. and A.T.A.). Where there was disagreement, another reviewer (O.A.I) was brought in to break the tie. At the end of the process, 15 eligible articles were selected for the final synthesis. The details of the Preferred Reporting Items for Systematic reviews and Meta-Analyses^16^ process for the selection are documented in Figure 1.

### Data extraction

The relevant qualitative and quantitative data were extracted from the selected articles using a piloted Microsoft Excel Worksheet software (Microsoft Corporation, Redmond, Washington, United States) individually by two investigators (T.O.O. and A.T.A.). Qualitative parameters extracted were the study design, location, region of Africa (based on regional grouping by the African Union; https://au.int/en/member_states/countryprofiles2), duration of study (in months), study time frame, sampled population, blood culture system, and the technique for bacteria identification and antibiotic susceptibility pattern of the bacteria associated with FN. The quantitative data extracted were the number of patients, the number of isolates obtained from the blood culture, the proportion of blood culture positivity, volume of blood sampled for microbiology investigation, antibiotic susceptibility, and patient outcome. Discrepancies in the data extracted were corrected by referring to the original studies.

### Data analysis

The data on isolates and antibiotic susceptibility patterns were aggregated from the selected articles and presented in proportions. Risk factors for FN were not included in this study, because of the varied study designs of the eligible articles with non-comparable study outcomes.

### Ethical considerations

This article followed all ethical standards for research without direct contact with human or animal subjects.

## Results

### Description of eligible articles

**FIGURE 2:**
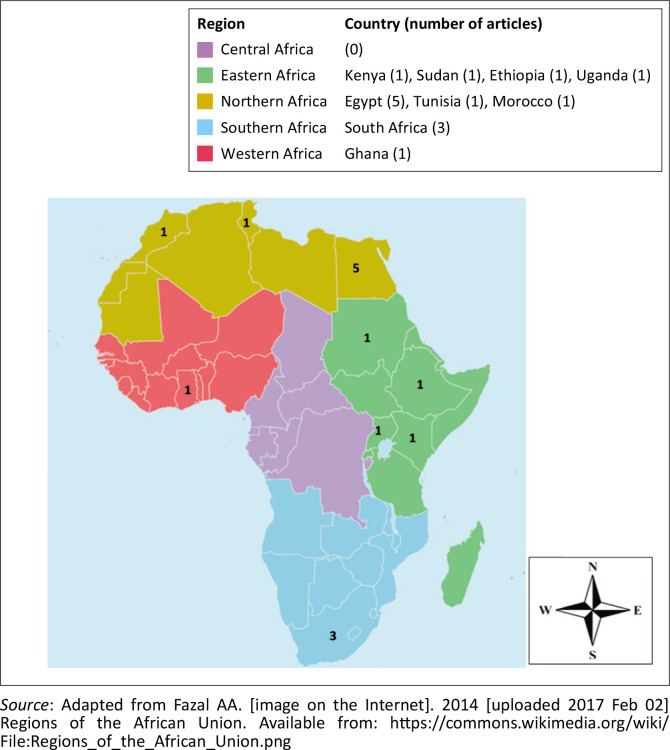
Distribution and number of eligible articles about febrile neutropenia in Africa (January 1991 to December 2024) across African Union Member States. Regions shown are as defined in the Organisation of African Union in 1976 (CM/Res. 464QCXVI).

The article search done in February 2024 and yielded a total of 16 637 articles from the three databases (Google Scholar [*n* = 16 500; PubMed [*n* = 103]; African Journals Online [*n* = 34]), of which 13 eligible original articles were selected for analysis. An additional two articles^17,18^ were included after a manual search of the references of the 13 eligible articles, resulting in a total of 15 articles for the synthesis after the selection criteria were applied. The 15 eligible studies were from nine countries, including Egypt (5), South Africa (3), Kenya (1), Sudan (1), Tunisia (1), Ghana (1), Morocco (1), Ethiopia (1), and Uganda (1) (Figure 2). The studies were conducted between 1991 and 2013 (Figure 2).

The majority of the studies were prospective (10 studies; 66.7%) (Supplementary Table 1), conducted at a single centre (14 studies; 93.3%) (data not shown in table). Many of the eligible articles had university teaching hospitals as study locations (seven studies; 46.7%). Four studies (26.7%) were from cancer institutes and specialist hospitals. Six studies (40%) were conducted among paediatric patients only, and among both paediatric and adult patients, while two studies (13.3%) were conducted among adult patients alone. Nine studies (60.0%) were conducted among a patient population with both haematological and solid tumours, and two studies (13.3%) were from a population of patients with stem cell transplantation.

### Medical microbiology laboratory methods

The medical microbiology laboratory methods used were stated in many of the studies; 10 studies stated the blood culture system used (automated blood culture system BACTEC/BACT ALERT, *n* = 7, and manual/conventional, *n* = 3). Ten studies stated bacterial identification methods used (conventional biochemical method only, *n* = 1; VITEK, *n* = 2; Phoenix, *n* = 1; Analytical Profile Index [API], *n* = 2; Sensititre, *n* = 1; Microscan, *n* = 2; BBL [Becton Dickinson Bioscience] Crystal ID, *n* = 1). Antibiotic susceptibility testing in 40% (6/11) of the studies was by disc diffusion methods (Supplementary Table 1).

### Bacteria agents

The review included 2062 patients, 2106 febrile episodes and a positive blood culture rate of 22.3% (1270/5675) (Table 1). A total of 1216 non-duplicate isolates were recovered from the selected studies, of which 57.0% (698/1225) were Gram-positive and 43.0% (527/1225) were Gram-negative isolates. The most common isolates were coagulase-negative *Staphylococcus* spp. (CONS; *n* = 281; 2.9%); *Staphylococcus aureus* (*n* = 153; 12.5%); *Klebsiella* spp. (*n* = 126; 10.3%); *Escherichia coli* (*n* = 99; 8.1%); *Streptococcus* spp. (*n* = 87; 7.1%); *Acinetobacter* spp. (*n* = 74; 6.0%) and *Pseudomonas* spp. (*n* = 69; 5.6%).

**TABLE 1:** Blood culture isolates from 15 studies on febrile neutropaenia in Africa, 1991–2024.

Variables	Total number (*n*)	Percentages (%)	References
**Total numbers**			
Total number of pooled patients from synthesised articles	2062	-	^1,19,20,21,22,23,24,25,26,27,28,29,30,31,32^
Total number of pooled bloodstream infection episodes from synthesised articles	2106	-	^1,19,20,21,22,23,24,25,26,27,28,29,30,32^
Total number of pooled culture-positive bacteraemia in patients with febrile neutropaenia from synthesised articles	1270/5675	22.3	^1,19,20,21,22,23,24,25,26,27,28,29,30,32^
**Total isolates**	1225	100.0	-
***Gram-positive isolates***	698	57.0	-
Coagulase-negative *Staphylococcus* spp.	281	22.9	^19,20,21,24,25,28,29,31,32^
Viridian group of *Streptococcus*	43	3.5	^22,24,29,30,32^
*Streptococcus* spp.	87	7.1	^19,20,25,26,27,31,32^
*Enterococcus* spp.	35	2.9	^20,22,25,28,29,30,32^
*Streptococcus pneumoniae*	21	1.7	^1,22,26,29,30,32^
*Staphylococcus* spp.	20	1.6	^19,26,27^
*Staphylococcus aureus*	153	12.5	^1,21,22,23,25,28,29,30,31,32^
*Aerococcus viridans*	4	0.3	^25^
*Rhodococcus* spp.	1	0.1	^23^
*Micrococcus* spp.	11	0.9	^25,31^
*Corynebacterium* spp.	3	0.2	^23,32^
*Listeria* spp.	1	0.1	^25^
*Bacillus* spp.	19	1.6	^23,29,30^
Gram-positive isolates not identified	19	1.6	^22,25,30,31^
***Gram-negative isolates***	527	43.0	-
*Escherichia coli*	99	8.1	^1,19,20,21,22,23,24,25,26,27,28,29,30,31,32^
*Klebsiella* spp.	126	10.3	^1,19,20,21,22,23,24,25,26,27,28,29,30,31,32^
*Enterobacter* spp.	38	3.1	^19,20,23,25,29,30,31,32^
*Flavimonas oryzihabitans*	1	0.1	^22^
*Cupriavidus pauculus*	1	0.1	^24^
*Citrobacter* spp.	12	1.0	^19,20,21,24,26,29^
*Serratia marcescens*	5	0.4	^19,29,31^
*Salmonella* spp.	9	0.7	^24,26,29,30^
*Proteus mirabilis*	7	0.6	^1,29^
*Acinetobacter* spp.	74	6.0	^19,20,21,22,23,24,25,26,27,29,30,31,32^
*Pseudomonas* spp.	69	5.6	^1,19,20,21,22,24,25,26,27,28,30,31,32^
*Yersinia* spp.	1	0.1	^31^
*Kluyvera ascorbata*	2	0.2	^31^
*Burkholderia cerpacia*	5	0.4	^21,30^
*Stenotrophomonas maltophilia*	12	1.0	^21,22,27,29,31^
*Chryseobacteria meningosepticum*	1	0.1	^31^
*Rolastonia picketti*	1	0.1	^31^
*Pasteurella* spp.	6	0.5	^25^
*Haemophilus influenzae*	3	0.2	^29^
*Moraxella* spp.	1	0.1	^29^
*Alcaligenes* spp.	2	0.2	^27^
*Ochtrobacterium* spp.	2	0.2	^27^
Gram-negative bacteria unidentified	50	4.1	^21,22,25,30^

Note: Please see full reference list of this article, Obadare TO, Adeyemo AT, Ibrahim OA, Sule NO, Adeyemo MM, Alatise OI. Bacterial agents and antibiotic resistance in febrile neutropaenia in Africa: A systematic review and meta-analysis. Afr J Lab Med. 2025;14(1), a2816. https://doi.org/10.4102/ajlm.v14i1.2816, for more information.

### Antibiotic susceptibility patterns

Antibiotic susceptibility patterns were reported in 11 studies (Supplementary Table 2). Of these studies, four reported the antibiotic susceptibility patterns based on the isolated organisms tested,^18,20,25,27^ four articles reported the antibiotic susceptibility based on Gram reactions of the isolated organisms,^17,19,24,28^ and three studies reported the proportion of resistance to tested antibiotics only without disaggregation-based isolated organisms.^22,23,29^ The overall rates of antibiotic resistance to antibiotics used for Gram-negative bacteria included ertapenem (1.9%; 1/52), meropenem (2.1%; 3/142), cefepime (32.8%; 141/430), piperacillin/tazobactam (40.9%; 133/325), tobramycin (4.8%; 3/63), ciprofloxacin (35.5%; 184/519), and colistin (12.5%; 7/56). The pulled antibiotic resistance rate for antibiotics used for Gram-positive bacteria included amoxicillin/clavulanic acid (71.8%; 163/227), vancomycin (2.8%; 7/321), clindamycin (50.7%; 37/73), and linezolid (2.8%; 1/30).

### Antibiotic resistance profile

For the most prevalent bacteria to the tested antibiotics, 66.7% (8/12) of CONS isolates were resistant to oxacillin/methicillin, mostly susceptible to vancomycin (resistance rate 5.3%; 2/38), and susceptible to linezolid (Table 2). In the same manner, *S. aureus* isolates were highly resistant to oxacillin/methicillin (68.8%; 22/32), and were mostly susceptible to vancomycin (10.0%; 1/10). *Klebsiella* spp. isolates were mostly resistant to ampicillin (93.8%; 15/16), piperacillin/tazobactam (85.7%; 6/7), and third-generation cephalosporins (75.9%; 22/29), and a quarter (4/16) showed resistance to the carbapenems. *Escherichia coli* showed similar high resistance to ceftazidime (72.7%; 8/11), and other third-generation cephalosporins (66.7%; 24/36). Non-fermenters such as *Acinetobacter* spp. showed resistance rates of between 50% and 100% to tested antibiotics, including gentamicin and ciprofloxacin. However, *Pseudomonas* spp. showed low resistance to ciprofloxacin (7.7%; 1/13).

**TABLE 2:** Antibiotic resistance to commonest isolates in febrile neutropaenia in Africa, January 1991 to December 2024.

Pathogens and relevant antibiotic	Number of isolates tested†	Number of isolates resistant	Proportion of resistance isolates (%)
**Coagulase-negative *Staphylococcus*** ^3,8,10^			
Ampicillin	40	6	15.0
Amoxycillin/clavulanic acid	40	7	17.5
Oxacillin/ methicillin	12	8	66.7
Cephalosporins (ceftriaxone/cefuroxime)	40	10	25.0
Tetracycline	13	5	38.5
Chloramphenicol	12	3	25.0
Linezolid	4	0	0.0
Vancomycin	38	2	5.3
Trimethoprim–sulfamethoxazole	22	2	9.1
***Staphylococcus aureus*** ^3,8,10^			
Penicillin	31	19	61.3
Ampicillin	34	15	44.1
Amoxycillin/clavulanic acid	32	20	62.5
Cephalosporins (ceftriaxone/cefuroxime)	35	23	65.7
Oxacillin/ methicillin	32	22	68.8
Tetracycline	34	14	41.2
Chloramphenicol	31	12	38.7
Vancomycin	10	1	10.0
Trimethoprim–sulfamethoxazole	32	13	40.6
***Klebsiella* spp.** ^20,21,22,23^			
Ampicillin	16	15	93.8
Amoxycillin/clavulanic acid	13	10	76.2
Cephalosporins (ceftriaxone/cefuroxime/cefotaxime)	29	22	75.9
Ceftazidime	10	7	70.0
Cefepime	3	0	0.0
Gentamycin	10	7	70.0
Quinolones (ciprofloxacin/levofloxacin/nalidixic acid)	18	9	50.0
Ertapenem/imipenem/meropenem	16	4	25.0
Trimethoprim–sulfamethoxazole	9	6	66.7
Piperacillin/tazobactam	7	6	85.7
***Escherichia coli*** ^20,21,22,23^			
Ampicillin	32	18	56.3
Amoxycillin/clavulanic acid	17	12	70.6
Cephalosporins (ceftriaxone/cefuroxime/cefotaxime)	36	24	66.7
Ceftazidime	11	8	72.7
Cefepime	4	0	0.0
Gentamycin	24	16	66.7
Quinolones (ciprofloxacin/levofloxacin/nalidixic acid)	23	14	60.9
Ertapenem/imipenem/meropenem	15	4	26.7
Trimethoprim–sulfamethoxazole	22	20	90.9
Piperacillin/tazobactam	15	6	40.0
***Acinetobacter* spp.** ^20,21^			
Gentamycin	7	6	85.7
Amikacin	7	4	57.1
Quinolones (ciprofloxacin/nalidixic acid)	4	2	50.0
Trimethoprim–sulfamethoxazole	7	7	100.0
***Pseudomonas* spp.** ^20,21^			
Quinolones (ciprofloxacin/levofloxacin)	13	1	7.7
Trimethoprim–sulfamethoxazole	1	1	100.0

Note: Please see full reference list of this article, Obadare TO, Adeyemo AT, Ibrahim OA, Sule NO, Adeyemo MM, Alatise OI. Bacterial agents and antibiotic resistance in febrile neutropaenia in Africa: A systematic review and meta-analysis. Afr J Lab Med. 2025;14(1), a2816. https://doi.org/10.4102/ajlm.v14i1.2816, for more information.

† , This reflects the cumulative number of isolates across cited studies with resistance to the antibiotics reported; not all studies tested resistance to the listed antibiotics.

## Discussion

Febrile neutropaenia is a medical emergency with attending consequences of morbidity, mortality and economic burden on both the patients and healthcare systems globally. The consequences of FN are further made difficult to deal with as the evolution of antimicrobial resistance escalates in LMICs amid a dilapidating healthcare system, complicated with layered drivers of antibiotic overuse and limited/absolute access to recommended newer antibiotics appropriate for treating MDR infections.^31^ While various international guides on the management of FN have been published, many LMICs are yet to develop their local guidelines. This may hamper the management of FN in these resource-constrained settings, where management of FN infections depends largely on empiric antibiotic therapy without recourse to the local epidemiological data and laboratory diagnostic capacity.

The burden of FN in Africa might not be fully described because of the paucity of data from this region. This review highlighted the low data output on FN compared to other regions, such as high-income countries, where oncology practice is more advanced, and is prioritised to be included in their health-benefit package for the public health sector, as there was no eligible study for this review in the last decade from Africa.^32^ In addition to this, there is skewness to the data on FN in Africa, as the North Africa region and East and Southern Africa were equally represented with seven eligible articles, and West and Central Africa region had one eligible article, which reflects on the quality of health investment and sophistication in healthcare systems available from these regions.^1^ This review also corroborates the already documented health disparity divide across the human development index that exists between high-income countries and LMICs in healthcare systems and deliveries.^32^ There is a need for more composite data on FN in Africa through research, funding, and improved transparent health systems, in addition to data infrastructures to bridge the cancer care inequalities.^1^

Blood cultures are a highly dependent test for the investigation of bloodstream infections, including FN in clinical microbiology laboratories.^33,34^ However, the procedure is limited by frequent contamination, as up to a third to half of all blood cultures are judged to be contaminants by infectious diseases physicians. The principal source of the contaminants is from the dermis commensals such as CONS, *Corynebacterium* spp., *Bacillus* spp, *Micrococcu*s spp., and *Cutibacterium* spp. because of poor disinfection of the skin.^35^ In this review, CONS (9 of 15 studies) accounted for over a fifth (23.1%) of the total isolates, which is similar to the contamination rate (23.0%) reported by Rupp et al.^36^ This may underscore the issue of contamination, which is a major problem in blood culture tests. While blood culture is the ‘gold standard’ for the investigation of blood stream infections, its accuracy hinges on effective compliance with the aseptic techniques through the pre- and analytical stages of the procedure (i.e. the selection of body site for sampling, aseptic cleaning of the venipuncture site, use of trained phlebotomist and downing of personal protective equipment).^35^ These underpin the need for specialised training for blood culture sample collection to improve the quality of diagnostic stewardship in the diagnoses of FN, isolating the pathogen involved to ensure targeted therapy for the treatment and improve treatment outcome in FN. Six out of the 10 eligible studies for this review made use of an automated blood culture system, while the others (four studies) used ‘manual’ blood culture systems. The ‘manual’ blood culture system reduces the culture yield of the investigation test and negatively impacts the promptness of the isolation, antibiotic susceptibility patterns, including timely and appropriate commencement of targeted therapy, as well as epidemiological surveillance of pathogens involved in FN.^34^ The uses of automated blood culture systems in Africa are usually restricted to major referral centres or reference laboratories, which are mostly powered by external collaboration from high-income countries. While the availability of the ‘automated’ blood culture system had significantly helped in the investigation of FN in LMICs of Africa, its sustainability remains in doubt after the funders’ collaboration because of the costly required regular equipment maintenance, complicated consumable logistics, and supply chain,^37^ as well as compatibility of this equipment with the tropical environment and infrastructural architecture.^1,34^

Clinical Laboratory Standards Institute and European Committee on Antimicrobial Susceptibility Testing guidelines on antibiotic susceptibility have stipulated specific antibiotics that are relevant for testing against specific bacteria. However, this was not followed in all cases. This could be because of the common use of a generic multidisc – a single-paneled antibiotic disc for Gram-negative and Gram-positive organisms, which might not accommodate the unique approach offered by a single antibiotic disc as stipulated by antibiotic susceptibility guidelines for the testing of organisms such as *Acinetobacter* spp, *Pseudomonas* spp. and *Streptococcus* spp.^38^ This review revealed that the clinical outcome of FN can be improved in Africa by increasing the diagnostic capacity of the laboratories infrastructures to isolate bacterial in bloodstream infection in FN, continuous training of the laboratory staff to improve their competencies to optimise blood culture testing, accurate pathogen identification, and antibiotic susceptibility testing to inform local policy development as well as adherence to international guidelines for comparable practice and result.^39^

The epidemiological distribution of bacteria in FN varies with geographical location, medical procedures, and antibiotic consumption in the locality. In this review, the significant variability across decades was not obvious. Gram-positive bacteria were the most isolated (56.7%) organism in this review, which is similar to the pattern documented over six years (1995–2000)^40^ in the United States, with a Gram-positive bacteria rate of 61.0% and a significant shift from Gram-negative to Gram-positive bacteria over two decades in Europe (1985–2000).^41^ This is in contrast to what was documented in Central America (from 2012 to 2014) where the most isolated group of bacteria in FN were Gram negative (78.0%),^42^ as well as in India (from 2012 to 2014; 57.8%) and Iran (from 2019 to 2020; 66.7%). The reasons for the predominance of Gram-positive bacteria in Africa in this review are unknown. However, it could be because of quinolone prophylaxis^43^ use in this setting, choice of mucositis-producing chemotherapy agents, as well as use of invasive devices such as central-line catheters and significant use of antacids.^44^

The use of prophylactic antibiotics (especially fluoroquinolone) has reduced the overall mortality in patients with an intermediate-to-high Multinational Association for Supportive Care in Cancer FN risk index. However, it is cautioned against by the European Organisation for Research and Treatment of Cancer and the American Society of Clinical Oncology, especially in cases with a low Multinational Association for Supportive Care in Cancer FN risk index (≥ 21), because of the risk of emergence of MDR.^45^ The aggregated resistance rate to ciprofloxacin and amoxycillin/clavulanic acid (which are the initial empiric antibiotics for low-risk patients with FN) from this review is more than a third (ciprofloxacin) and about two-quarters (amoxycillin/clavulanic acid) of the isolates, and implies that these antibiotics might not be effective empiric therapy for FN in many Africa settings because of the prevailing high antibiotic resistance situation driven by increasing antibiotic consumption in the hospital and community, as support for a failing, fractured heath system and antibiotic stewardship intervention.^31,46^ The use of cefepime and piperacillin/tazobactam as a monotherapy or as part of combined antibiotics for initial empiric therapy in high-risk patients with FN needs caution when escalating for treatment of FN, as two-fifths of the isolates were resistant to piperacillin/tazobactam and a third to cefepime. This underscores the need for optimised medical microbiology support for appropriate blood culture, pathogen isolation and determination of antibiotic susceptibility patterns to guide the escalation and de-escalation of antibiotic use in FN.^47,48^ Commonly isolated Gram-negatives in FN in this review, such as *Klebsiella* spp, and *E. coli,* had high resistance to piperazilin/tazobactam, third-generation cephalosporins, and even last-result drugs such as carbapenems. In addition, there is a high prevalence of methicillin resistance in Gram-positive cocci; however, vancomycin is still effective, as only about 10% of *S. aureus* isolates, and about 20% of CONS, are resistant to vancomycin. The pattern of antibiotic resistance distributed across the spectrum of bacterial pathogens isolated in the FN from this review revealed that antibiotic resistance, especially to last-resort antibiotics such as carbapenems in Gram-negative bacilli organisms, is more widespread. This is a huge concern that the MDR organisms are increasingly being implicated in FN, and this poses a significant challenge to treatment in such patients. In the light of this, especially with increasing morbidity and mortality because of carbapenem resistance in Enterobacterales and non-glucose-fermenting Gram-negative bacilli, there is an increasing role in the use of beta-lactams (and non-beta-lactams) in combination with beta-lactam-inhibitors such as ceftazidime-avibactam, ceftolozane-tazobactam, imipenem/relebactam, and others. There is, however, a challenge regarding access to this newer combination of antibiotics in LMICs because of their high cost, and limited data on their use for FN in such climes.^46^ Therefore, it is imperative to prioritise prevention of infection in patients with neutropaenia by modification of intensity of cytotoxic medication, addition of myeloid-growth-factor, systematic selection of patients for chemoprophylaxis, colonisation surveillance for MDR, and proper infection prevention and control.^46,47,49^

### Limitations

There is an inherent limitation with systematic review, as its outcome is based on an aggregation of the secondary data from synthesised original articles. It was observed that some eligible articles did not follow the antibiotic susceptibility guidelines to select the antibiotics tested on the bacterial agents. Moreover, in some cases, the breakpoints, the panels of antibiotics for some bacteria, had changed over time. These made the articles heterogeneous and presented challenges to getting a common denominator for the antibiotic susceptibility results for some analyses. However, these limitations did not nullify the essence of this study to report the situation analysis on the state of FN in Africa in the context of antibiotic resistance.

### Conclusion

This review highlighted the paucity of data on FN in Africa, which corroborated the health disparity in cancer treatment across the economic divide of the nations. Our findings underscore the need for good laboratory infrastructures as well as correct blood culture technique in investigating FN to ensure isolation of true pathogens and appropriate ‘drug-bug’ combinations based on standard antibiotic susceptibility guidelines to reflect the appropriate epidemiological picture for the development of local guidelines in managing FN in Africa. There is a high level of MDR strains among the organisms isolated in FN in this review, which makes a case for continuous need for antimicrobial resistance surveillance, improved laboratory capacity and stewardship. There is also a need for the review of international guidelines and suggested antibiotic prophylaxis and empiric therapy for FN in the light of local epidemiological findings. There might be a need for improved access to newer agents, such as beta-lactam-beta-lactamase inhibitors, in Africa to treat FN caused by MDR, causing resistance to last-resort antibiotics (such as carbapenems) for improved outcomes in FN patients. This review should encourage further studies on bacterial agents of FN in this clime to generate needed pieces of evidence to support improvement in medical microbiology laboratory capacity for the diagnosis of infections in FN to inform local empirical guidelines and definitive therapy for optimised management of cancer patients.
